# 9-(2-Thien­yl)-9*H*-carbazole

**DOI:** 10.1107/S1600536808037173

**Published:** 2008-11-26

**Authors:** Xu-Liang Jiang, Feng-Rong Li, Ren-Hua Zheng

**Affiliations:** a School of Pharmaceutical Engineering, Shenyang Pharmaceutical University, Shenyang, 110016, People’s Republic of China; b School of Pharmaceutical and Chemical Engineering, Taizhou University, Linhai, 317000, People’s Republic of China

## Abstract

In the title compound, C_16_H_11_NS, the dihedral angles between the fused ring system and the pendant thienyl ring are 86.37 (5) and 57.14 (5)°.

## Related literature

For the fluorescence properties of 9-(2-thien­yl)-9*H*-carbazole and its application in organic electroluminescent devices, including flat-panel displays, see: Wu *et al.* (2001 ▶).
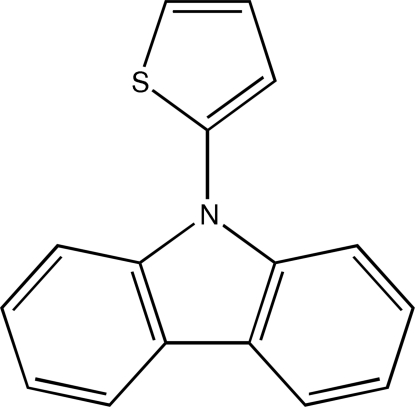


## Experimental

### 

#### Crystal data

C_16_H_11_NS
*M* *_r_* = 249.33
Monoclinic, 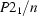

*a* = 14.412 (3) Å
*b* = 9.5831 (19) Å
*c* = 18.671 (4) Å
β = 100.64 (3)°
*V* = 2534.4 (9) Å^3^
*Z* = 8
Mo *K*α radiation
μ = 0.23 mm^−1^
*T* = 298 (2) K
0.25 × 0.20 × 0.15 mm

#### Data collection

Bruker SMART APEX CCD diffractometer
Absorption correction: multi-scan (*SADABS*; Bruker, 2000 ▶) *T*_min_ = 0.94, *T*_max_ = 0.97
26852 measured reflections
4980 independent reflections
4138 reflections with *I* > 2σ(*I*)
*R*_int_ = 0.035
3 standard reflections frequency: 60 min intensity decay: 0.3%

#### Refinement

*R*[*F* ^2^ > 2σ(*F* ^2^)] = 0.040
*wR*(*F* ^2^) = 0.080
*S* = 1.13
4980 reflections
325 parameters
H-atom parameters constrained
Δρ_max_ = 0.11 e Å^−3^
Δρ_min_ = −0.13 e Å^−3^

### 

Data collection: *SMART* (Bruker, 2000 ▶); cell refinement: *SAINT* (Bruker, 2000 ▶); data reduction: *SAINT*; program(s) used to solve structure: *SHELXS97* (Sheldrick, 2008 ▶); program(s) used to refine structure: *SHELXL97* (Sheldrick, 2008 ▶); molecular graphics: *ORTEP-3 for Windows* (Farrugia, 1997 ▶); software used to prepare material for publication: *WinGX* (Farrugia, 1999 ▶).

## Supplementary Material

Crystal structure: contains datablocks I, global. DOI: 10.1107/S1600536808037173/sg2270sup1.cif

Structure factors: contains datablocks I. DOI: 10.1107/S1600536808037173/sg2270Isup2.hkl

Additional supplementary materials:  crystallographic information; 3D view; checkCIF report

## Figures and Tables

**Fig. 1.**
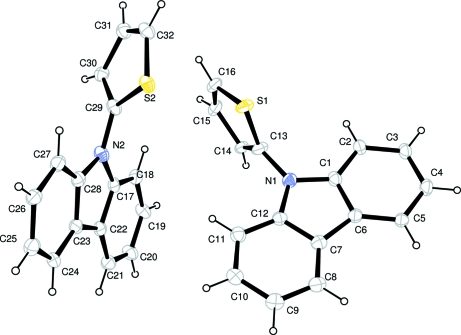
The structure of (I), showing the atom-labelling scheme. Ellipsoids are drawn at the 30% probability level.

## supplementary crystallographic information

### Comment

Due to its excellent fluorescence properties, 9-(2-thienyl)-9*H*-carbazole can be used in organic electroluminescent devices, which have received considerable attention for their potential application in flat-panel displays (Wu *et al.*, 2001). It was readily synthesized *via* Ullmann reaction with copper(I) iodide as catalyst from carbazole and 2-iodothiophene.

There are two crystallographically independent molecules in the sturcture of (I). The independent molecula is built up from  a central core containing three fused rings and one pendant five-membered ring. (Fig. 1). In two independent molecules, the three fused rings are coplanar within 0.0493 (15) and 0.0135 (15) Å, respectively. The five-membered rings are coplanar within 0.0062 (13) and 0.0173 (12) Å, respectively. The dihedral angles between the two components are 86.37 (5) and 57.14 (5)°, respectively.

### Experimental

The title compound was synthesized *via* Ullmann reaction with copper(I) iodide as catalyst from carbazole and 2-iodothiophene. A solution of the compound in ethanol was concentrated gradually at room temperature to afford colorless prisms.

### Refinement

H atoms were included in calculated positions and refined using a riding model. H atoms were given isotropic displacement parameters equal to 1.2 times the equivalent isotropic displacement parameters of their parent atoms and C—H distances were restrained to 0.93 Å.

### Crystal data

**Table:**

C_16_H_11_NS	*F*_000_ = 1040
*M**_r_* = 249.33	*D*_x_ = 1.307 Mg m^−^^3^
Monoclinic, *P*2_1_/*n*	Mo *K*α radiation λ = 0.71073 Å
Hall symbol: -P 2yn	Cell parameters from 7198 reflections
*a* = 14.412 (3) Å	θ = 2.1–23.5º
*b* = 9.5831 (19) Å	µ = 0.23 mm^−^^1^
*c* = 18.671 (4) Å	*T* = 298 (2) K
β = 100.64 (3)º	Prismatic, colorless
*V* = 2534.4 (9) Å^3^	0.25 × 0.20 × 0.15 mm
*Z* = 8	

### Data collection

**Table:**

Bruker SMART APEX CCD diffractometer	4980 independent reflections
Radiation source: fine-focus sealed tube	4138 reflections with *I* > 2σ(*I*)
Monochromator: graphite	*R*_int_ = 0.035
*T* = 298(2) K	θ_max_ = 26.0º
ω/2θ scans	θ_min_ = 2.0º
Absorption correction: multi-scan(SADABS; Bruker, 2000)	*h* = −17→17
*T*_min_ = 0.94, *T*_max_ = 0.97	*k* = −11→11
26852 measured reflections	*l* = −23→22

### Refinement

**Table:**

Refinement on *F*^2^	Secondary atom site location: difference Fourier map
Least-squares matrix: full	Hydrogen site location: inferred from neighbouring sites
*R*[*F*^2^ > 2σ(*F*^2^)] = 0.040	H-atom parameters constrained
*wR*(*F*^2^) = 0.080	*w* = 1/[σ^2^(*F*_o_^2^) + (0.0269*P*)^2^ + 0.456*P*] where *P* = (*F*_o_^2^ + 2*F*_c_^2^)/3
*S* = 1.13	(Δ/σ)_max_ < 0.001
4980 reflections	Δρ_max_ = 0.11 e Å^−^^3^
325 parameters	Δρ_min_ = −0.12 e Å^−^^3^
Primary atom site location: structure-invariant direct methods	Extinction correction: none

### Special details

**Table:**

Geometry. All e.s.d.'s (except the e.s.d. in the dihedral angle between two l.s. planes) are estimated using the full covariance matrix. The cell e.s.d.'s are taken into account individually in the estimation of e.s.d.'s in distances, angles and torsion angles; correlations between e.s.d.'s in cell parameters are only used when they are defined by crystal symmetry. An approximate (isotropic) treatment of cell e.s.d.'s is used for estimating e.s.d.'s involving l.s. planes.
Refinement. Refinement of *F*^2^ against ALL reflections. The weighted *R*-factor *wR* and goodness of fit *S* are based on *F*^2^, conventional *R*-factors *R* are based on *F*, with *F* set to zero for negative *F*^2^. The threshold expression of *F*^2^ > σ(*F*^2^) is used only for calculating *R*-factors(gt) *etc*. and is not relevant to the choice of reflections for refinement. *R*-factors based on *F*^2^ are statistically about twice as large as those based on *F*, and *R*- factors based on ALL data will be even larger.

### Fractional atomic coordinates and isotropic or equivalent isotropic displacement parameters (Å^2^)

**Table:**

	*x*	*y*	*z*	*U*_iso_*/*U*_eq_	
C1	0.63934 (12)	0.55497 (19)	0.16359 (10)	0.0479 (4)	
C2	0.66476 (13)	0.5659 (2)	0.09552 (10)	0.0528 (4)	
H2	0.6505	0.4940	0.0618	0.063*	
C3	0.71148 (12)	0.68426 (19)	0.07780 (10)	0.0516 (4)	
H3	0.7285	0.6916	0.0323	0.062*	
C4	0.73279 (13)	0.7917 (2)	0.12815 (11)	0.0578 (5)	
H4	0.7640	0.8709	0.1163	0.069*	
C5	0.70737 (12)	0.78082 (19)	0.19622 (10)	0.0522 (4)	
H5	0.7216	0.8527	0.2299	0.063*	
C6	0.66065 (12)	0.66244 (19)	0.21394 (9)	0.0497 (4)	
C7	0.62079 (12)	0.62015 (18)	0.27751 (9)	0.0472 (4)	
C8	0.61740 (14)	0.6816 (2)	0.34441 (10)	0.0566 (5)	
H8	0.6464	0.7674	0.3563	0.068*	
C9	0.57060 (13)	0.6150 (2)	0.39353 (11)	0.0547 (5)	
H9	0.5683	0.6562	0.4383	0.066*	
C10	0.52720 (13)	0.4869 (2)	0.37576 (10)	0.0545 (5)	
H10	0.4959	0.4423	0.4086	0.065*	
C11	0.53059 (13)	0.4254 (2)	0.30887 (10)	0.0543 (5)	
H11	0.5015	0.3397	0.2970	0.065*	
C12	0.57739 (12)	0.49203 (18)	0.25974 (9)	0.0449 (4)	
C13	0.55629 (13)	0.3243 (2)	0.15350 (10)	0.0550 (5)	
C14	0.61217 (12)	0.19052 (17)	0.15100 (9)	0.0451 (4)	
H14	0.6738	0.1687	0.1728	0.054*	
C15	0.53843 (13)	0.1030 (2)	0.10260 (11)	0.0568 (5)	
H15	0.5507	0.0114	0.0908	0.068*	
C16	0.45484 (13)	0.1629 (2)	0.07748 (10)	0.0541 (5)	
H16	0.4063	0.1168	0.0469	0.065*	
C17	0.35787 (12)	0.10050 (16)	0.29912 (9)	0.0425 (4)	
C18	0.43576 (12)	0.06295 (18)	0.26924 (10)	0.0497 (4)	
H18	0.4295	0.0512	0.2191	0.060*	
C19	0.52295 (13)	0.0429 (2)	0.31425 (9)	0.0519 (4)	
H19	0.5751	0.0178	0.2943	0.062*	
C20	0.53225 (13)	0.0605 (2)	0.38914 (10)	0.0549 (5)	
H20	0.5906	0.0471	0.4193	0.066*	
C21	0.45436 (12)	0.09801 (18)	0.41902 (10)	0.0490 (4)	
H21	0.4606	0.1097	0.4691	0.059*	
C22	0.36717 (12)	0.11803 (17)	0.37402 (9)	0.0458 (4)	
C23	0.27331 (13)	0.15467 (16)	0.38691 (9)	0.0448 (4)	
C24	0.23654 (13)	0.18710 (19)	0.44865 (9)	0.0487 (4)	
H24	0.2754	0.1857	0.4943	0.058*	
C25	0.14168 (13)	0.22164 (19)	0.44213 (11)	0.0527 (4)	
H25	0.1171	0.2433	0.4834	0.063*	
C26	0.08358 (13)	0.22374 (19)	0.37386 (10)	0.0523 (4)	
H26	0.0201	0.2468	0.3695	0.063*	
C27	0.12036 (12)	0.19131 (19)	0.31212 (10)	0.0506 (4)	
H27	0.0815	0.1927	0.2664	0.061*	
C28	0.21522 (12)	0.15678 (16)	0.31864 (9)	0.0448 (4)	
C29	0.23207 (12)	0.12673 (18)	0.18875 (10)	0.0472 (4)	
C30	0.23683 (11)	0.00776 (19)	0.13801 (9)	0.0462 (4)	
H30	0.2601	−0.0819	0.1486	0.055*	
C31	0.19480 (12)	0.06941 (19)	0.06602 (10)	0.0520 (4)	
H31	0.1909	0.0190	0.0230	0.062*	
C32	0.16284 (13)	0.2012 (2)	0.06608 (10)	0.0522 (4)	
H32	0.1326	0.2472	0.0244	0.063*	
N1	0.58992 (10)	0.45050 (15)	0.19114 (8)	0.0485 (3)	
N2	0.26435 (10)	0.12467 (15)	0.26517 (8)	0.0474 (3)	
S1	0.44448 (4)	0.32622 (5)	0.10522 (3)	0.05735 (14)	
S2	0.18295 (3)	0.27280 (5)	0.14807 (3)	0.04925 (12)	

### Atomic displacement parameters (Å^2^)

**Table:**

	*U*^11^	*U*^22^	*U*^33^	*U*^12^	*U*^13^	*U*^23^
C1	0.0430 (9)	0.0521 (10)	0.0493 (10)	−0.0014 (8)	0.0103 (8)	0.0063 (8)
C2	0.0550 (11)	0.0525 (11)	0.0504 (10)	−0.0083 (8)	0.0080 (8)	0.0116 (8)
C3	0.0468 (10)	0.0556 (11)	0.0541 (11)	−0.0092 (8)	0.0139 (8)	0.0104 (9)
C4	0.0483 (10)	0.0566 (11)	0.0669 (12)	−0.0148 (9)	0.0068 (9)	0.0136 (9)
C5	0.0470 (10)	0.0510 (10)	0.0509 (10)	−0.0087 (8)	−0.0109 (8)	0.0109 (8)
C6	0.0436 (10)	0.0505 (10)	0.0493 (10)	−0.0047 (8)	−0.0063 (8)	0.0082 (8)
C7	0.0417 (9)	0.0472 (10)	0.0497 (10)	0.0078 (7)	0.0009 (8)	−0.0004 (8)
C8	0.0611 (12)	0.0503 (11)	0.0543 (11)	0.0072 (9)	−0.0002 (9)	−0.0043 (9)
C9	0.0551 (11)	0.0513 (10)	0.0591 (11)	0.0177 (9)	0.0145 (9)	−0.0086 (9)
C10	0.0494 (10)	0.0579 (11)	0.0565 (11)	0.0104 (9)	0.0106 (8)	0.0058 (9)
C11	0.0545 (11)	0.0531 (11)	0.0552 (11)	0.0009 (9)	0.0097 (9)	0.0028 (9)
C12	0.0432 (9)	0.0464 (9)	0.0437 (9)	0.0021 (7)	0.0042 (7)	−0.0016 (7)
C13	0.0532 (11)	0.0535 (11)	0.0506 (10)	−0.0036 (9)	−0.0102 (8)	−0.0099 (8)
C14	0.0453 (9)	0.0463 (10)	0.0418 (9)	−0.0069 (7)	0.0028 (7)	−0.0079 (7)
C15	0.0508 (11)	0.0561 (11)	0.0653 (12)	−0.0188 (9)	0.0152 (9)	−0.0135 (9)
C16	0.0502 (11)	0.0537 (11)	0.0577 (11)	−0.0153 (9)	0.0081 (9)	−0.0165 (9)
C17	0.0510 (10)	0.0284 (8)	0.0482 (9)	−0.0020 (7)	0.0090 (8)	0.0080 (7)
C18	0.0463 (10)	0.0475 (10)	0.0557 (11)	−0.0032 (8)	0.0106 (8)	0.0046 (8)
C19	0.0548 (11)	0.0571 (11)	0.0465 (10)	0.0114 (9)	0.0166 (8)	0.0084 (8)
C20	0.0501 (11)	0.0657 (12)	0.0470 (10)	0.0090 (9)	0.0042 (8)	0.0149 (9)
C21	0.0503 (10)	0.0445 (9)	0.0490 (10)	0.0020 (8)	0.0005 (8)	0.0069 (8)
C22	0.0542 (10)	0.0345 (8)	0.0469 (9)	−0.0040 (7)	0.0048 (8)	0.0101 (7)
C23	0.0635 (11)	0.0276 (8)	0.0439 (9)	−0.0045 (7)	0.0119 (8)	−0.0024 (7)
C24	0.0535 (11)	0.0554 (11)	0.0399 (9)	−0.0126 (8)	0.0158 (8)	0.0018 (8)
C25	0.0532 (11)	0.0464 (10)	0.0600 (11)	−0.0042 (8)	0.0139 (9)	−0.0044 (8)
C26	0.0523 (11)	0.0565 (10)	0.0499 (10)	−0.0134 (9)	0.0138 (8)	−0.0097 (8)
C27	0.0465 (10)	0.0527 (10)	0.0539 (11)	−0.0106 (8)	0.0126 (8)	−0.0072 (8)
C28	0.0532 (10)	0.0303 (8)	0.0515 (10)	−0.0011 (7)	0.0116 (8)	−0.0006 (7)
C29	0.0502 (10)	0.0431 (9)	0.0494 (10)	0.0099 (8)	0.0118 (8)	0.0140 (7)
C30	0.0322 (8)	0.0503 (10)	0.0541 (10)	0.0011 (7)	0.0030 (7)	0.0012 (8)
C31	0.0471 (10)	0.0538 (11)	0.0555 (11)	−0.0005 (8)	0.0106 (8)	0.0027 (9)
C32	0.0498 (10)	0.0581 (11)	0.0494 (10)	0.0133 (8)	0.0107 (8)	0.0146 (8)
N1	0.0513 (8)	0.0442 (8)	0.0500 (8)	−0.0015 (7)	0.0098 (7)	−0.0030 (6)
N2	0.0490 (8)	0.0439 (8)	0.0501 (8)	0.0089 (6)	0.0112 (7)	0.0135 (6)
S1	0.0547 (3)	0.0552 (3)	0.0540 (3)	−0.0029 (2)	−0.0115 (2)	−0.0119 (2)
S2	0.0514 (3)	0.0445 (2)	0.0527 (3)	0.0103 (2)	0.0120 (2)	0.0151 (2)

### Geometric parameters (Å, °)

**Table:**

C1—N1	1.382 (2)	C17—C18	1.390 (2)
C1—C2	1.390 (2)	C17—C22	1.390 (2)
C1—C6	1.390 (3)	C17—N2	1.399 (2)
C2—C3	1.390 (2)	C18—C19	1.390 (3)
C2—H2	0.9300	C18—H18	0.9300
C3—C4	1.390 (3)	C19—C20	1.390 (2)
C3—H3	0.9300	C19—H19	0.9300
C4—C5	1.390 (3)	C20—C21	1.390 (2)
C4—H4	0.9300	C20—H20	0.9300
C5—C6	1.390 (2)	C21—C22	1.390 (2)
C5—H5	0.9300	C21—H21	0.9300
C6—C7	1.468 (2)	C22—C23	1.460 (2)
C7—C8	1.390 (2)	C23—C24	1.390 (2)
C7—C12	1.390 (2)	C23—C28	1.390 (2)
C8—C9	1.390 (3)	C24—C25	1.390 (3)
C8—H8	0.9300	C24—H24	0.9300
C9—C10	1.390 (3)	C25—C26	1.390 (3)
C9—H9	0.9300	C25—H25	0.9300
C10—C11	1.390 (3)	C26—C27	1.390 (2)
C10—H10	0.9300	C26—H26	0.9300
C11—C12	1.390 (2)	C27—C28	1.390 (2)
C11—H11	0.9300	C27—H27	0.9300
C12—N1	1.385 (2)	C28—N2	1.361 (2)
C13—N1	1.437 (2)	C29—N2	1.417 (2)
C13—C14	1.519 (3)	C29—C30	1.492 (2)
C13—S1	1.6950 (19)	C29—S2	1.6849 (17)
C14—C15	1.515 (2)	C30—C31	1.491 (2)
C14—H14	0.9300	C30—H30	0.9300
C15—C16	1.339 (3)	C31—C32	1.344 (2)
C15—H15	0.9300	C31—H31	0.9300
C16—S1	1.6638 (19)	C32—S2	1.654 (2)
C16—H16	0.9300	C32—H32	0.9300
			
N1—C1—C2	129.48 (17)	C19—C18—H18	120.0
N1—C1—C6	110.47 (15)	C17—C18—H18	120.0
C2—C1—C6	120.00 (17)	C18—C19—C20	120.00 (17)
C1—C2—C3	120.00 (18)	C18—C19—H19	120.0
C1—C2—H2	120.0	C20—C19—H19	120.0
C3—C2—H2	120.0	C21—C20—C19	120.00 (17)
C2—C3—C4	120.00 (17)	C21—C20—H20	120.0
C2—C3—H3	120.0	C19—C20—H20	120.0
C4—C3—H3	120.0	C20—C21—C22	120.00 (17)
C5—C4—C3	120.00 (17)	C20—C21—H21	120.0
C5—C4—H4	120.0	C22—C21—H21	120.0
C3—C4—H4	120.0	C21—C22—C17	120.00 (17)
C6—C5—C4	120.00 (18)	C21—C22—C23	134.05 (17)
C6—C5—H5	120.0	C17—C22—C23	105.94 (15)
C4—C5—H5	120.0	C24—C23—C28	120.00 (17)
C5—C6—C1	120.00 (17)	C24—C23—C22	134.21 (17)
C5—C6—C7	134.17 (18)	C28—C23—C22	105.78 (15)
C1—C6—C7	105.77 (15)	C25—C24—C23	120.00 (17)
C8—C7—C12	120.00 (17)	C25—C24—H24	120.0
C8—C7—C6	133.90 (18)	C23—C24—H24	120.0
C12—C7—C6	106.07 (15)	C24—C25—C26	120.00 (18)
C7—C8—C9	120.00 (18)	C24—C25—H25	120.0
C7—C8—H8	120.0	C26—C25—H25	120.0
C9—C8—H8	120.0	C27—C26—C25	120.00 (18)
C8—C9—C10	120.00 (18)	C27—C26—H26	120.0
C8—C9—H9	120.0	C25—C26—H26	120.0
C10—C9—H9	120.0	C28—C27—C26	120.00 (17)
C11—C10—C9	120.00 (18)	C28—C27—H27	120.0
C11—C10—H10	120.0	C26—C27—H27	120.0
C9—C10—H10	120.0	N2—C28—C27	128.75 (16)
C12—C11—C10	120.00 (18)	N2—C28—C23	111.24 (15)
C12—C11—H11	120.0	C27—C28—C23	120.00 (16)
C10—C11—H11	120.0	N2—C29—C30	125.71 (14)
N1—C12—C11	129.82 (17)	N2—C29—S2	119.87 (13)
N1—C12—C7	110.18 (15)	C30—C29—S2	114.42 (13)
C11—C12—C7	120.00 (16)	C31—C30—C29	102.18 (15)
N1—C13—C14	126.32 (15)	C31—C30—H30	128.9
N1—C13—S1	116.91 (14)	C29—C30—H30	128.9
C14—C13—S1	116.74 (12)	C32—C31—C30	116.99 (17)
C15—C14—C13	99.68 (14)	C32—C31—H31	121.5
C15—C14—H14	130.2	C30—C31—H31	121.5
C13—C14—H14	130.2	C31—C32—S2	112.91 (14)
C16—C15—C14	117.26 (17)	C31—C32—H32	123.5
C16—C15—H15	121.4	S2—C32—H32	123.5
C14—C15—H15	121.4	C1—N1—C12	107.48 (14)
C15—C16—S1	114.91 (14)	C1—N1—C13	125.31 (15)
C15—C16—H16	122.5	C12—N1—C13	127.19 (15)
S1—C16—H16	122.5	C28—N2—C17	107.05 (14)
C18—C17—C22	120.00 (16)	C28—N2—C29	128.08 (15)
C18—C17—N2	130.01 (16)	C17—N2—C29	124.64 (15)
C22—C17—N2	109.98 (15)	C16—S1—C13	91.39 (9)
C19—C18—C17	120.00 (17)	C32—S2—C29	93.42 (9)
			
N1—C1—C2—C3	177.18 (17)	C28—C23—C24—C25	0.0 (2)
C6—C1—C2—C3	0.0 (3)	C22—C23—C24—C25	178.35 (17)
C1—C2—C3—C4	0.0 (3)	C23—C24—C25—C26	0.0 (3)
C2—C3—C4—C5	0.0 (3)	C24—C25—C26—C27	0.0 (3)
C3—C4—C5—C6	0.0 (3)	C25—C26—C27—C28	0.0 (3)
C4—C5—C6—C1	0.0 (3)	C26—C27—C28—N2	−178.69 (17)
C4—C5—C6—C7	−176.78 (18)	C26—C27—C28—C23	0.0 (2)
N1—C1—C6—C5	−177.68 (15)	C24—C23—C28—N2	178.91 (15)
C2—C1—C6—C5	0.0 (3)	C22—C23—C28—N2	0.13 (18)
N1—C1—C6—C7	−0.1 (2)	C24—C23—C28—C27	0.0 (2)
C2—C1—C6—C7	177.60 (16)	C22—C23—C28—C27	−178.77 (15)
C5—C6—C7—C8	−2.1 (3)	N2—C29—C30—C31	−179.27 (16)
C1—C6—C7—C8	−179.20 (19)	S2—C29—C30—C31	0.93 (17)
C5—C6—C7—C12	176.08 (19)	C29—C30—C31—C32	−2.7 (2)
C1—C6—C7—C12	−1.02 (19)	C30—C31—C32—S2	3.4 (2)
C12—C7—C8—C9	0.0 (3)	C2—C1—N1—C12	−176.23 (18)
C6—C7—C8—C9	177.97 (18)	C6—C1—N1—C12	1.2 (2)
C7—C8—C9—C10	0.0 (3)	C2—C1—N1—C13	2.3 (3)
C8—C9—C10—C11	0.0 (3)	C6—C1—N1—C13	179.69 (16)
C9—C10—C11—C12	0.0 (3)	C11—C12—N1—C1	178.44 (18)
C10—C11—C12—N1	179.68 (17)	C7—C12—N1—C1	−1.85 (19)
C10—C11—C12—C7	0.0 (3)	C11—C12—N1—C13	0.0 (3)
C8—C7—C12—N1	−179.74 (16)	C7—C12—N1—C13	179.66 (16)
C6—C7—C12—N1	1.78 (19)	C14—C13—N1—C1	85.2 (2)
C8—C7—C12—C11	0.0 (3)	S1—C13—N1—C1	−92.9 (2)
C6—C7—C12—C11	−178.48 (16)	C14—C13—N1—C12	−96.6 (2)
N1—C13—C14—C15	−178.93 (18)	S1—C13—N1—C12	85.3 (2)
S1—C13—C14—C15	−0.78 (19)	C27—C28—N2—C17	178.81 (17)
C13—C14—C15—C16	1.1 (2)	C23—C28—N2—C17	0.03 (18)
C14—C15—C16—S1	−1.1 (2)	C27—C28—N2—C29	4.2 (3)
C22—C17—C18—C19	0.0 (2)	C23—C28—N2—C29	−174.56 (15)
N2—C17—C18—C19	−179.34 (17)	C18—C17—N2—C28	179.21 (17)
C17—C18—C19—C20	0.0 (3)	C22—C17—N2—C28	−0.19 (17)
C18—C19—C20—C21	0.0 (3)	C18—C17—N2—C29	−6.0 (3)
C19—C20—C21—C22	0.0 (3)	C22—C17—N2—C29	174.63 (15)
C20—C21—C22—C17	0.0 (2)	C30—C29—N2—C28	−125.31 (19)
C20—C21—C22—C23	178.93 (18)	S2—C29—N2—C28	54.5 (2)
C18—C17—C22—C21	0.0 (2)	C30—C29—N2—C17	61.0 (2)
N2—C17—C22—C21	179.46 (15)	S2—C29—N2—C17	−119.22 (16)
C18—C17—C22—C23	−179.20 (14)	C15—C16—S1—C13	0.45 (17)
N2—C17—C22—C23	0.27 (17)	N1—C13—S1—C16	178.60 (16)
C21—C22—C23—C24	2.2 (3)	C14—C13—S1—C16	0.28 (16)
C17—C22—C23—C24	−178.76 (17)	C31—C32—S2—C29	−2.25 (16)
C21—C22—C23—C28	−179.28 (18)	N2—C29—S2—C32	−179.18 (15)
C17—C22—C23—C28	−0.24 (17)	C30—C29—S2—C32	0.64 (14)

## References

[bb1] Bruker (2000). *CAD-4 EXPRESS* *SMART*, *SAINT* and *SADABS* Bruker AXS Inc., Madison, Wisconsin, USA.

[bb2] Farrugia, L. J. (1997). *J. Appl. Cryst.* **30**, 565.

[bb3] Farrugia, L. J. (1999). *J. Appl. Cryst.* **32**, 837–838.

[bb6] Sheldrick, G. M. (2008). *Acta Cryst.* A**64**, 112–122. 10.1107/S0108767307043930 18156677

[bb7] Wu, I.-Y., Lin, J. T., Tao, Y.-T., Balasubramaniam, E., Su, Y. Z. & Ko, C.-W. (2001). *Chem. Mater.* **13**, 2626–2631.

